# Decrease in Population and Increase in Welfare of Community Cats in a Twenty-Three Year Trap-Neuter-Return Program in Key Largo, FL: The ORCAT Program

**DOI:** 10.3389/fvets.2019.00007

**Published:** 2019-02-01

**Authors:** Rachael E. Kreisler, Heather N. Cornell, Julie K. Levy

**Affiliations:** ^1^Pathology and Population Medicine, Midwestern University College of Veterinary Medicine, Glendale, AZ, United States; ^2^Maddie's Shelter Medicine Program, University of Florida, Gainesville, FL, United States

**Keywords:** trap-neuter-return, TNR, free-roaming cats, feral cats, stray cats, community cats, animal welfare, retrovirus

## Abstract

The objective of this study was to evaluate the effect of a long-term (23-year) trap-neuter-return program on the population size of community cats in the Ocean Reef Community and to describe the demographic composition and outcome of enrolled cats. A retrospective study was performed using both cat census data collected between 1999 and 2013 as well as individual medical records for cats whose first visit occurred between 3/31/1995 and 12/31/2017. Medical record entries were reviewed to determine program inputs, cat outcomes, retroviral disease prevalence, and average age of first visit, sterilization, and death through 6/11/2018. Change over time was analyzed via linear regression. The free-roaming population decreased from 455 cats recorded in 1999 to 206 recorded in 2013 (55% decrease, *P* < 0.0001). There were 3,487 visits recorded for 2,529 community cats, with 869 ovariohysterectomies and 822 orchiectomies performed. At last recorded visit, there were 1,111 cats returned back to their original location, and 1,419 cats removed via adoption (510), transfer to the adoption center (201), euthanasia of unhealthy or retrovirus positive cats (441), died in care (58), or outcome of dead on arrival (209). The number of first visits per year decreased 80% from 348 in 1995 to 68 in 2017. The estimated average age of the active cat population increased by 0.003 months each year (*P* = 0.031) from 16.6 months in 1995 to 43.8 months in 2017. The mean age of cats at removal increased 1.9 months per year over time (*P* < 0.0001) from 6.4 months in 1995 to 77.3 months in 2017. The mean age of cats at return to the original location was 20.8 months, which did not change over time. The overall retrovirus prevalence over the entire duration was 6.5%, with FIV identified in 3.3% of cats and FeLV identified in 3.6%. Retrovirus prevalence decreased by 0.32% per year (*P* = 0.001), with FIV decreasing by 0.16% per year (*P* = 0.013) and FeLV decreasing 0.18% per year (*P* = 0.033). In conclusion, a trap-neuter-return program operating for over two decades achieved a decrease in population and an increase in population welfare as measured by increased average age of population and decreased retrovirus prevalence.

## Introduction

Trap Neuter Return (TNR) programs exist in large part to reduce population size and growth rate by decreasing reproduction (1–5). Reductions in population size are desirable due to concerns regarding wildlife predation, public health and nuisance factors (6). In addition to reducing population size or growth, TNR is also promoted as a method for improving cat welfare (3, 4, 7–10). TNR of free-roaming cats may decrease predation as compared to populations that are not sterilized or provided anthropogenic food sources (11). TNR allows for the provision of veterinary care, including vaccination against infectious disease, treatment of injuries and illnesses, and humane euthanasia for animals found to be suffering. It is also a method for promoting humane communities by avoiding euthanasia as a means of population control or nuisance abatement.

Multiple studies have shown TNR to be effective in reducing population size or curtailing population growth, but they are complicated by the fact that many colonies are not geographically restricted (2, 4, 12–14). The presence of a long-term TNR program with both population level and detailed individual information was a unique opportunity to study the impacts of sustained TNR on a geographically isolated population of free-roaming cats.

The objective of this study was to evaluate the effect of a long-term (23 year) TNR program on the population size of community cats in the Ocean Reef Community and to describe the demographic composition and outcome of cats enrolled in the TNR program. These findings can be used by shelters and other invested parties to estimate the impact of TNR on cat welfare and provide input parameters for mathematical models used to estimate the impact of TNR programs on community cat populations.

## Materials and Methods

### Study Community

The community of Ocean Reef occupies ~2,500 acres on the northernmost tip of Key Largo in the Florida Keys. It is a peninsula approximately four miles long and a mile wide, with a single gated road staffed 24 h a day leading into the community. This private club is bordered on three sides by water and on the fourth by protected state and federal conservation land. Ocean Reef contains ~1,700 homes, although much of the occupation is seasonal and there is a correspondingly large number of seasonal workers.^1^

Five unaltered cats were brought to Ocean Reef by a groundskeeper to perform rat control in the 1960s. While the cats controlled the rat problem successfully, by the 1980s, the number of cats had grown large enough to be themselves considered a nuisance to the increasing number of residents. Over 2,000 cats are stated anecdotally to have been present in the 1980s. Population control measures, which included lethal methods, were instituted to control the cat population. As an alternative to lethal measures, an individual resident began to trap cats and bring them to a local veterinarian for neutering. In 1995, the Ocean Reef Community Association (ORCA) supported the opening of a spay/neuter clinic in Ocean Reef and the formation of the ORCAT program to provide sterilization, care, and feeding to the free-roaming cats (15). In 2006, the Grayvik Animal Care Center opened, which contains a full-service veterinary and grooming clinic for the pets of residents in addition to a cat adoption center and sanctuary. There has been a single individual in the role of director of the ORCAT program since its inception, maintaining feeding stations, creating individual cat medical records and performing episodic surveys of the population. This position reports to the Vice President of Ocean Reef and is accountable for annual goals. Only two veterinarians have been the main provider of services for the population, one from 1995 to 1998, and the other since 1998.

Surveys of the cat population were performed between 1999 and 2013. Documented population surveys were not executed after 2013, although cats continued to be cared for and TNR efforts continued. Surveys were recorded by marking feeding stations on a paper map and recording the total number of cats per feeding station. The number and location of feeding stations was determined by homeowner preference, convenience, and minimization of feeding station colony size. The initial number of feeding stations was large in order to facilitate complete trapping of colonies, which was easier with smaller numbers of cats per colony, and to minimize fighting between cats. All cat counts were performed by the caretaker.

Cats were trapped when un-marked individuals were noted at feeding stations, or when previously sterilized cats required veterinary care. Individual medical records for each cat were maintained in paper files. Each cat's visit (check-in to check-out at the medical center) was documented in the medical record. At their first visit, cats were routinely neutered, marked by ear-tipping, vaccinated with FVRCP, rabies and FeLV vaccines, and dewormed (pyrantel pamoate, praziquantel). They were also tested for FIV antibodies and FeLV antigen;^2^ cats that tested positive for either retrovirus were typically euthanized prior to administration of routine preventive care. Cats were determined to be euthanized for retrovirus status if they were euthanized concurrently with a positive test and there was no evidence that the cat was otherwise significantly unhealthy. A date of birth was estimated through the joint effort of the caretaker and veterinarian. Upon re-trapping, cats were provided with vaccine boosters for FVRCP, rabies and FeLV and medical care as required. Microchipping of cats was implemented beginning in mid-2005.

### Study Design

A retrospective study was performed using both aggregate cat census data spanning years 1999–2013 as well as review of individual cat medical records for cats whose first visit occurred from 3/31/1995 through 12/31/2017. Feeding stations and their associated populations were geocoded to visualize the change in population over time through Geographic Information System mapping technology.^3^ Geographic changes were visualized via hexbin maps in order to protect privacy. The paper-based medical records were coded and entered into a custom database.^4^ The associated cat demographics and outcomes were used to generate descriptive statistics and graphs.^5^

For population-level analyses based on individual records (estimated count, average age, and age structure of population) a likely date of death was calculated for each cat with an outcome of returned. The estimated date of death was determined by calculating the mean age for cats at outcome which had an outcome of DOA or euthanasia. This was compared to their age at return, and if younger, the difference was calculated and added to their date of return to determine a likely date of death. If older, an additional 12 months was added to the likely date of death. The data for the population-level analyses was then constructed by creating a scaffold consisting of each day contained within the study period and performing an outer join with the individual records to select cats with a date of birth less than or equal to the scaffold date and a date of death (or estimated death) greater than or equal to the scaffold date. Average age of the cat population per year was determined by calculating the age of each cat per year between birth and removal by death or likely death which included euthanasia, died in care, dead on arrival (DOA) or missing in action (MIA). The status of MIA was assigned to cats that had not been sighted at their usual feeding station for an unusual period of time, as determined by the caretaker. Cats removed from the active population by adoption were not included in the average age analysis. Linear regression was used to analyze change over time.^6^ Significance was set at *p* < 0.05 for all quantitative analyses.

## Results

### Population of Cats

**Figure 1:**
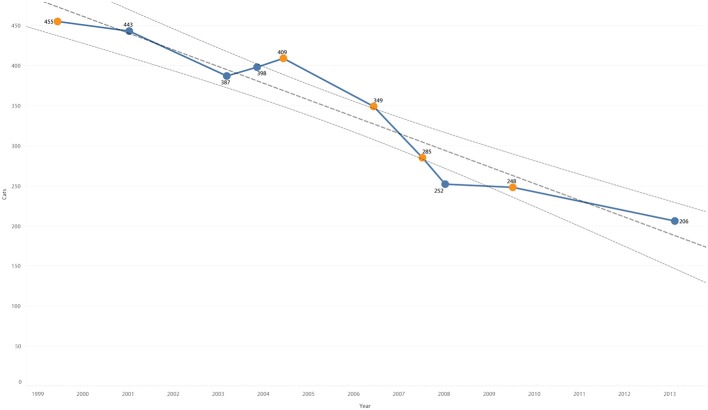
Cat population from census by year overlaid with trend line and 95% confidence interval. Summer months in orange, winter months in blue.

Surveys of the cat population occurred in June 1999, January 2001, March 2003, November 2003, June 2004, June 2006, July 2007, January 2008, July 2009, and February 2013. Per the census records, the free-roaming cat population decreased over time from 455 cats recorded in 1999 to 206 recorded in 2013 (55% decrease). The decrease was linear and significant, with a slope of −0.06, *P* < 0.0001 (Figure 1). Neither month of the year nor a binary seasonal variable of fall/winter as compared to spring/summer were significant.

**Figure 2:**
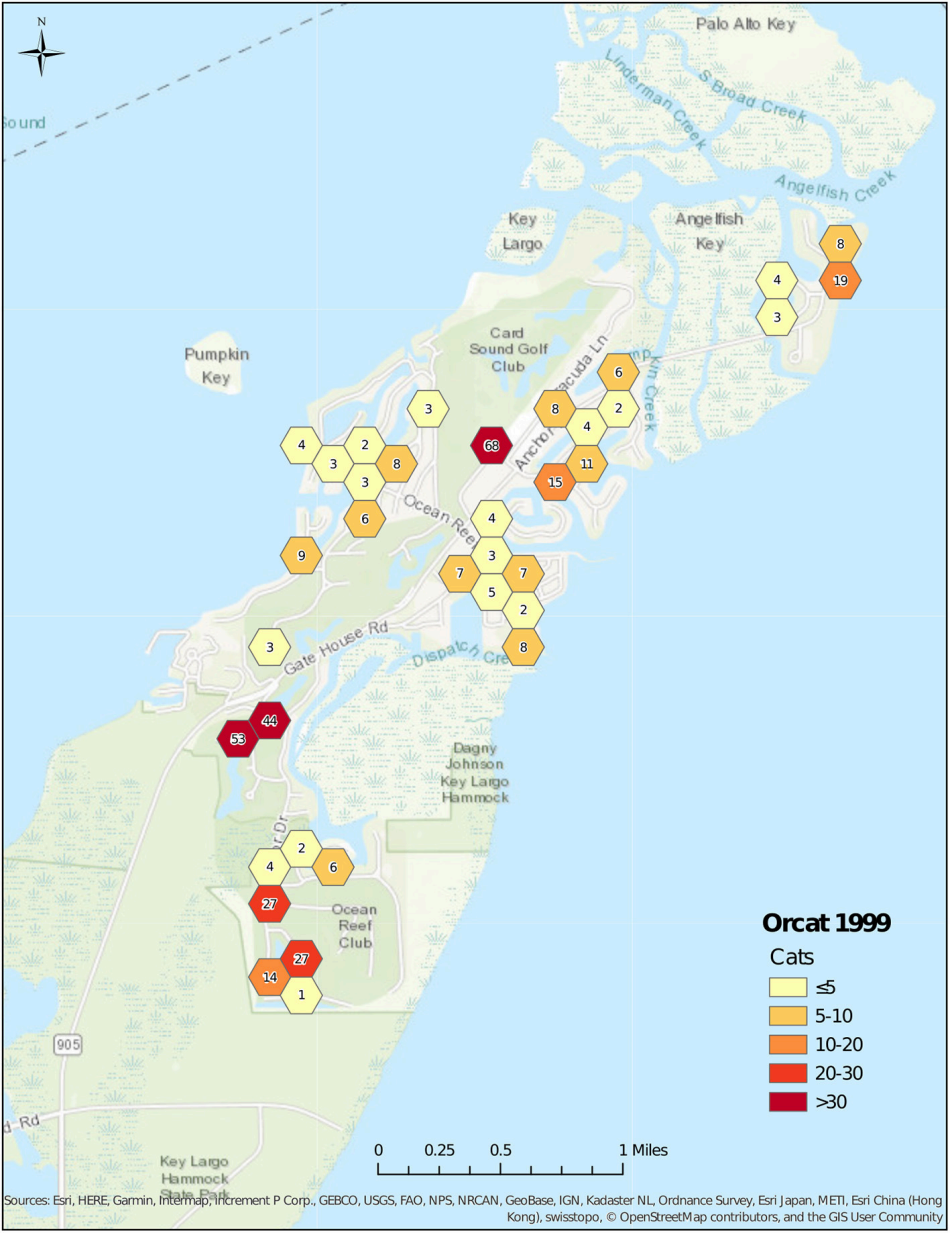
Cat census locations, 1999.

**Figure 3:**
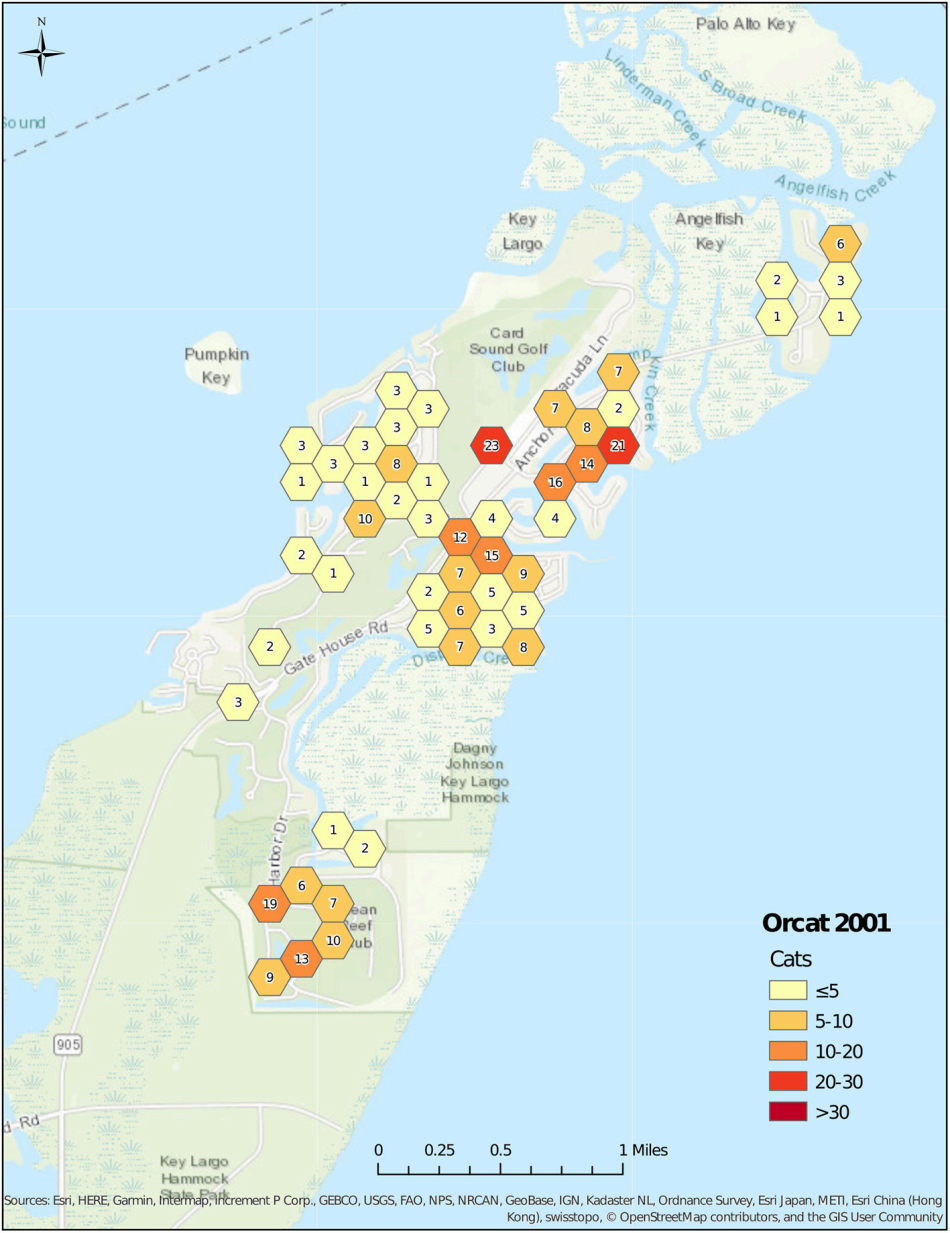
Cat census locations, 2001.

**Figure 4:**
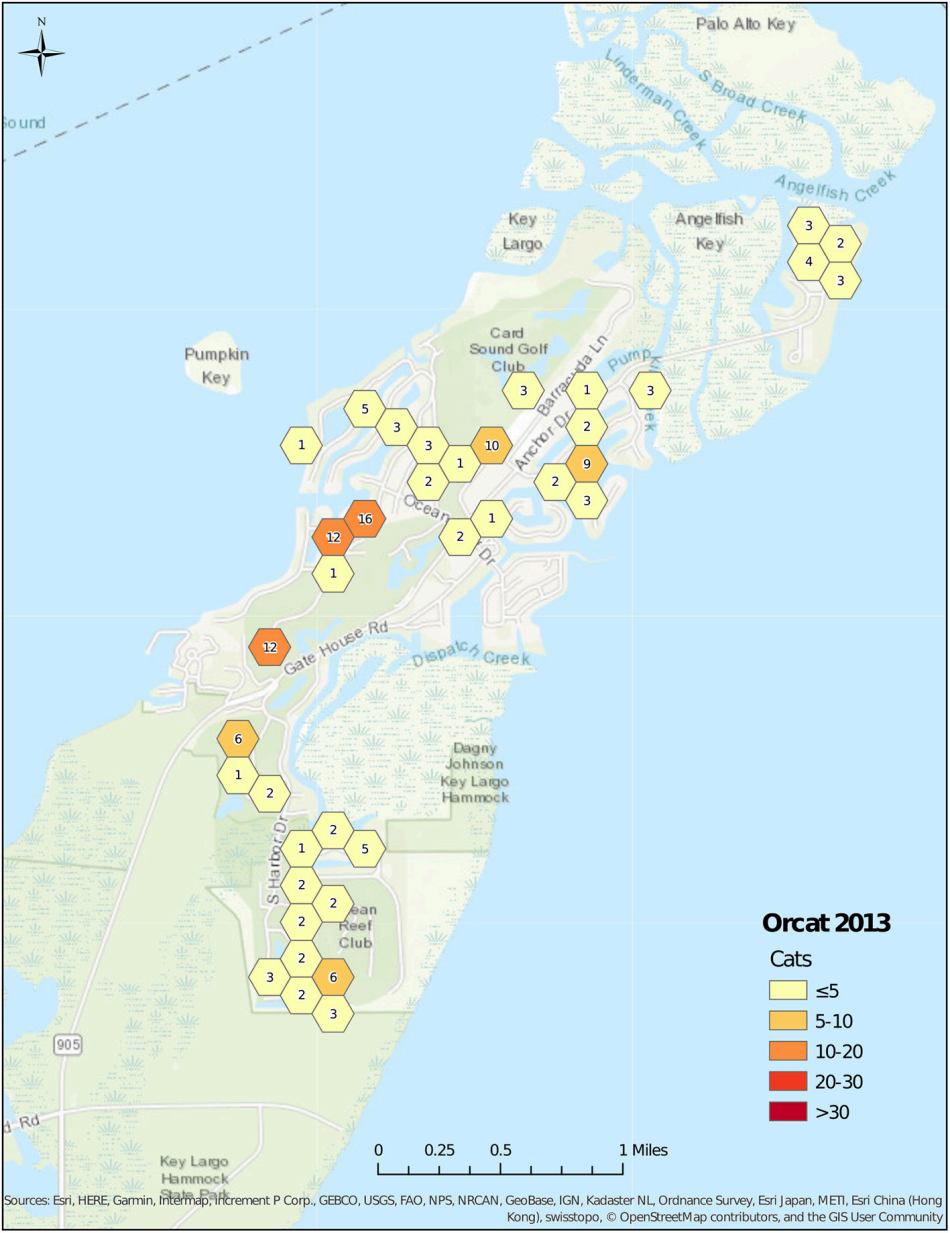
Cat census locations, 2013.

The number of feeding stations changed over time, starting with 60 stations in 1999 (Figure 2), and increasing to 85 stations in 2001 (Figure 3). Stations were maintained at a number between 76 and 82 until 2008, and then decreased to 44 in 2013 (Figure 4). The average number of cats per station started at 7.6 in 1999, decreased to 5.2 in 2001, was maintained at between 4.6 and 5.3 from 2001 to 2006, before decreasing to 3.1 in 2008. After 2008, the average number of cats per station increased to 4.7 in 2013 as the number of feeding stations decreased more rapidly than did the number of cats.

### Individual Records

**Figure 5:**
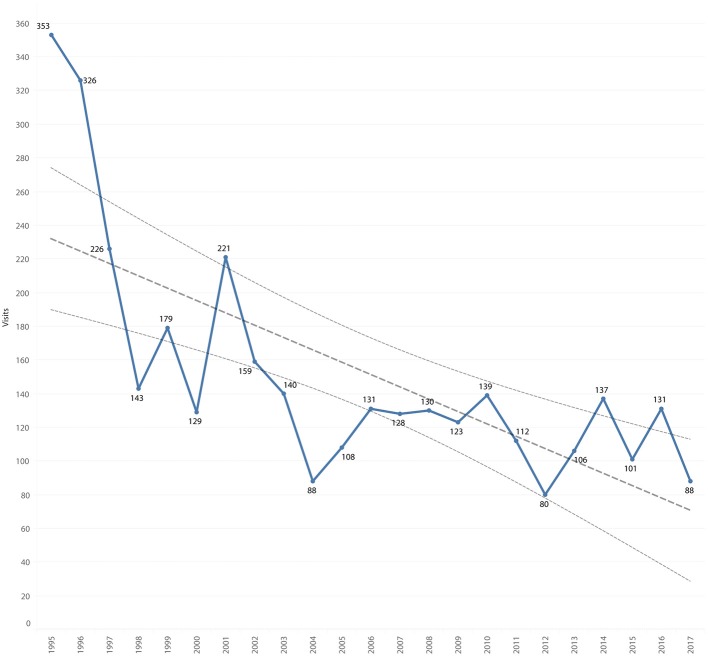
All visits by year, overlaid with trend line and 95% confidence interval.

**Figure 6:**
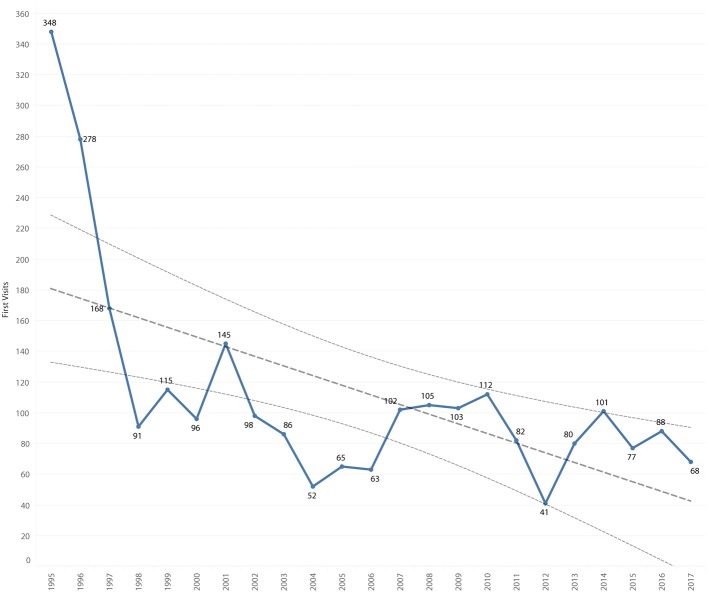
First visits by year, overlaid with trend line and 95% confidence interval.

There were 3,487 visits to the clinic recorded for 2,571 records of 2,529 community cats. There was a mean of 1.4 visits per cat, with 1,995 (77.6%) cats having only a single visit. Of the 2,571 records, 119 (4.6%) were missing an estimated date of birth, 19 (0.7%) a gender and 42 (1.6%) were suspected to be a duplicate of a prior identification number. The number of clinic visits decreased 75.1% from 353 in 1995 to 88 in 2017 (Figure 5). The greatest decrease occurred between 1995 and 2004, with a decrease of 23.3 visits per year (*P* = 0.004). After 2004, the mean number of visits was 116.5 per year, and there was no significant difference in the number of visits between years 2005 and 2017. First visits decreased 80.5% (Figure 6) from 348 in 1995 to 68 in 2017. The mean number of first visits was 111.5 (range 41–348). First visits fell sharply from 348 in 1995 to 52 in 2004, with a decrease of 25.5 first visits per year (*P* = 0.004). After 2004, there was a mean of 83.6 first visits per year, which did not change significantly between 2005 and 2017.

### Program Inputs

A total of 1,691 gonadectomies were performed, including 869 ovariohysterectomies and 822 orchiectomies. Over 18% of cats (479) were found to be already sterilized at their first visit, whether from sterilization prior to the official ORCAT program started in 1995, duplicate cats, trapping efforts by individuals or from lost/abandoned cats. Of the cats found to be already sterilized, 196 (40.9%) were also previously ear tipped; however, 13 of these ear tipped cats were noted to not be ORCAT's. An additional 165 non-sterilization surgeries were performed to treat injuries. A total of 2,327 FeLV, 1,897 rabies, and 2,727 FVRCP vaccines were administered. Over 2,800 fecal examinations were completed, and 2,327 FIV/FeLV tests were performed. Of female cats undergoing ovariohysterectomy, 11.5% were pregnant, with a mean of 4 fetuses (range 1–6).

### Retroviral Prevalence

**Figure 7:**
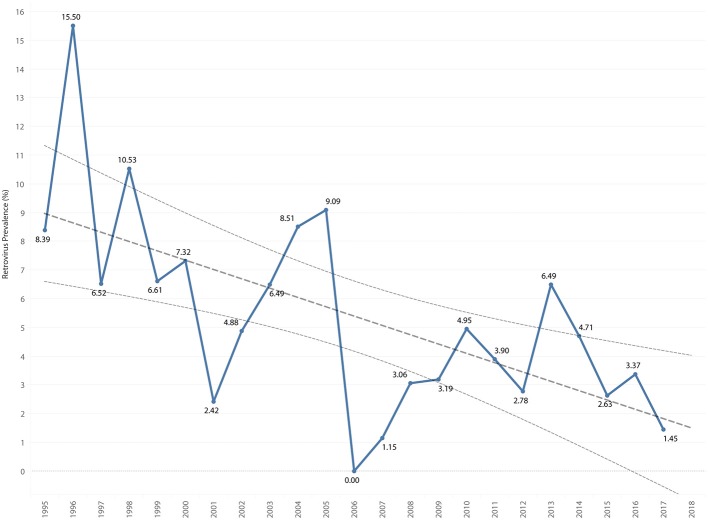
Total retrovirus prevalence by year overlaid with trend line and 95% confidence interval.

**Figure 8:**
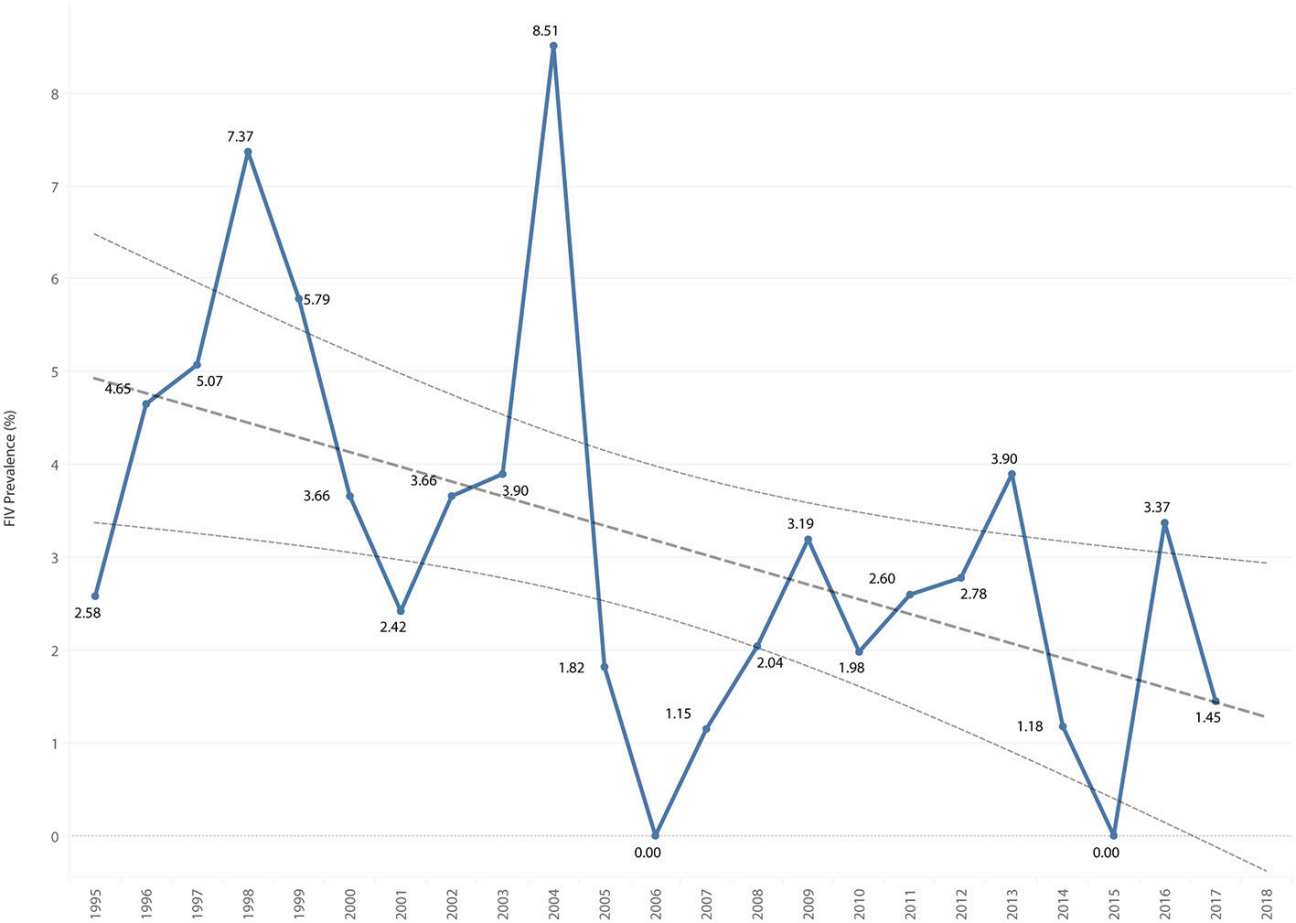
FIV prevalence by year overlaid with trend line and 95% confidence interval.

**Figure 9:**
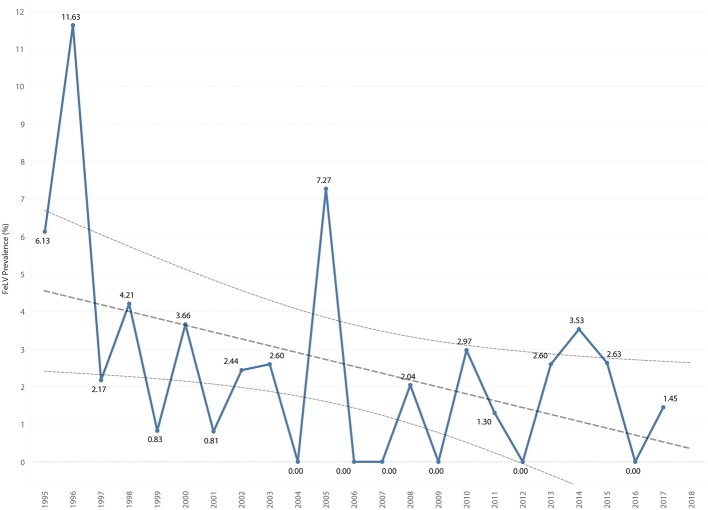
FeLV prevalence by year overlaid with trend line and 95% confidence interval.

The overall retrovirus seropositivity was 6.5%, with 9 cats positive for both FIV and FeLV. The overall prevalence of FIV was 3.3%, with a range of 0.0–8.5% per year. The overall prevalence of FeLV was 3.6%, with a range of 0.0–11.6% per year. Total retrovirus prevalence decreased by 0.32% per year (*P* = 0.001), Figure 7. FIV prevalence decreased by 0.16% per year (*P* = 0.013), Figure 8. FeLV prevalence decreased 0.18% per year (*P* = 0.033), Figure 9.

### Cat Outcomes

**Figure 10:**
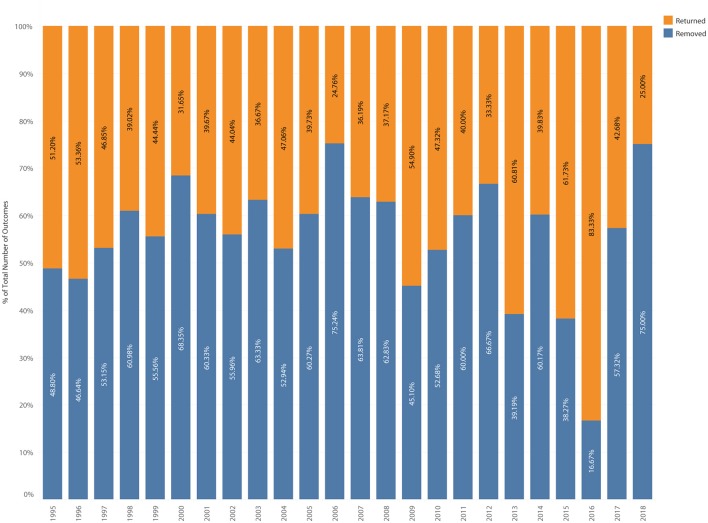
Last visit outcome category of removed or returned as percent of total outcomes.

**Figure 11:**
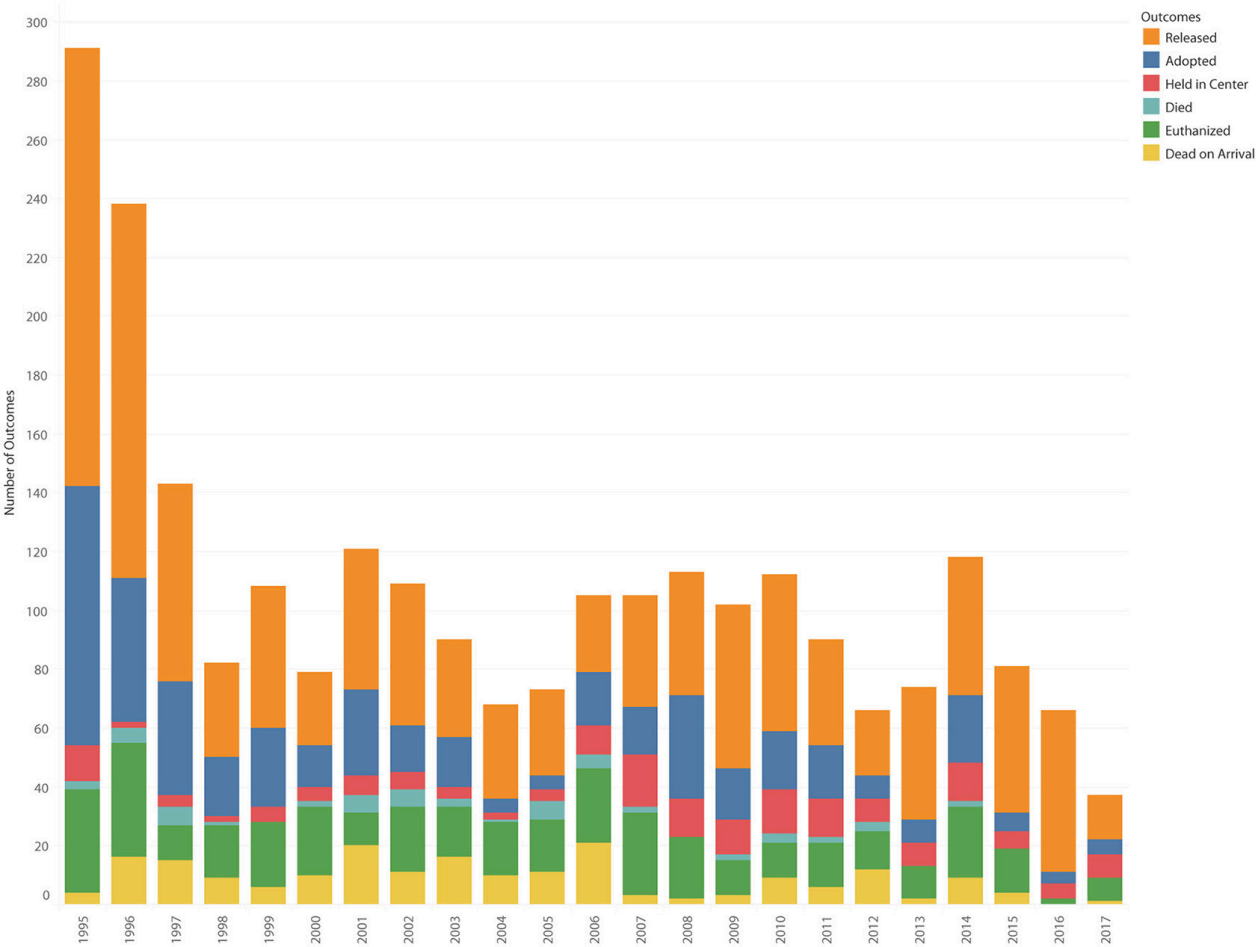
Last visit outcomes by outcome type.

Outcomes for visits were classified as either returned or removed, with an average of 50.0% (range 16.7–83.3%) of visits ending in removal per year (Figure 10). Removal included adoption, transfer to Grayvik center, died in care, euthanasia, and DOA, while returned included outcomes of released and missing in action (MIA). Of the 1,869 visits ending in release, 318 (17.0%) released the cat to a different location than they had been trapped due to a conflict with the original location. For the final disposition (outcome of the last recorded visit), 1,111 cats were released back to their outdoor location, and 1,419 cats were removed via adoption (510), transfer to the adoption center (201), died in care (58), euthanasia of unhealthy or retrovirus-positive cats (441), or outcome of DOA (209), Figure 11. Six of 9 (67%) cats were euthanized for double-positive retrovirus status, 61 of 73 (84%) for FeLV positive status and 45 of 67 (67%) for FIV positive status, with the remainder of the euthanized cats, 329 (75%), euthanized due to health. Cats that were DOA had cause of death split between trauma (43.1%), unknown (43.1%), trapped in fumigation tent (9.1%), and illness (4.8%). Trauma was primarily from motor vehicles (81.1%), unknown (10.0%), and predation (8.9%).

### Estimated Age Structure and Sex Distribution

**Figure 12:**
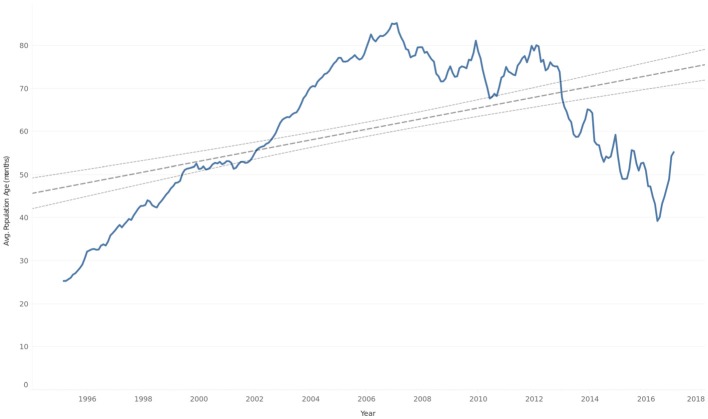
Average age (months) of cat population by quarter overlaid with trend line and 95% confidence interval.

**Figure 13:**
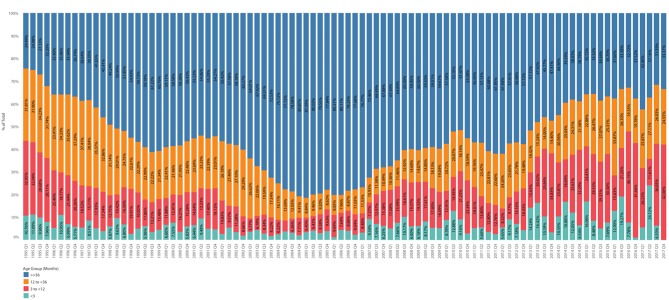
Age structure by age group as percent of total population by quarter.

The mean estimated age of cats at first visit was 21 months (95%CI 20 to 23), with a range of 0 (newborn) through 275 months. For cats sexually intact at first visit (2,026), the mean age was 11 months (95%CI 10 to 11), with a range of 0 through 204 months. For cats already sterilized and ear-tipped at first visit, the mean age was 70.3 months (95%CI 62.5 to 78.2), with a range of 6.7–204 months. For cats already sterilized, but with no documented ear-tip, the mean age at first visit was similar to previously sterilized cats with an ear-tip at 76.4 months (95%CI 69.1 to 83.6), with a range of 2.0–275 months. For previously sterilized cats, the age at first visit increased by 0.01 months per year (*P* = 0.043). There was no change over time in the age of cats intact at first visit. The estimated average age (calculated age of cats without an outcome of removed) of the active cat population increased by 0.003 months each year (*P* = 0.030; Figure 12). The estimated age structure fluctuated over time (Figure 13).

Overall, the mean age of cats at removal was 41.3 months (95%CI 38.2 to 44.4), which increased 1.9 months per year (*P* < 0.0001). The mean age at adoption was 11.3 months (95%CI 9.2–13.5), which did not change significantly over time. The mean age at euthanasia was 82.1 months (95%CI 75.3 to 88.8) which increased over time by 4.0 months per year (*P* < 0.0001). The mean age of DOA/MIA cats was 58.7 (95%CI 51.2 to 66.2) which increased by 1.4 months per year (*P* = 0.028). The mean age of died in care was 36.2 (95%CI 20.8 to 51.5) which did not change over time. The mean age of cats removed to the Grayvik center was 20.9 months (95%CI 15.4 to 26.5) which increased by 1.3 months per year (*P* = 0.004). The mean age of released cats was 20.4 (95%CI 18.7 to 22.1) which did not change over time. The mean age of cats euthanized for double retrovirus positive status was 3.7 years, while it was 4.1 and 2.3 for FIV and FeLV, respectively. Age at euthanasia for positive retrovirus status did not change over time. Double retrovirus positive cats not euthanized at time of diagnosis survived a mean of 13.3 months (95%CI 0 to 36.2) after diagnosis, with all double positive cats having an ultimate outcome of euthanasia. Cats positive for FIV not euthanized at time of diagnosis and with a final outcome of died, euthanized or DOA survived a mean of 15.4 months (95%CI 2.9 to 27.9) while cats similarly positive for FeLV survived a mean of 7.1 months (95%CI 0 to 19.3).

Females accounted for 52% (95%CI 49.7 to 53.6) of the population at first visit. The mean age of females at first visit was 22.9 (95%CI 20.7 to 25.1), while it was 19.4 (95%CI 17.4 to 21.3) for males. Females that were intact at first visit had a mean age of 11.0 months (95%CI 9.8 to 12.1), while males intact at first visit had a mean age of 9.8 (95%CI 8.7 to 10.9). Females that were previously sterilized were the oldest at first visit with a mean of 79.3 months of age (95%CI 71.5 to 87.2), with males that were previously sterilized having a mean of 67.5 months of age (95%CI 60.1 to 74.9). Females had a mean age of 32.7 months (95%CI 30.0 to 35.5) at last visit, while males had a mean age of 31.0 (95%CI 28.3 to 33.6). Females intact at first visit had an age at last visit of 21.1 months (95%CI 18.9 to 23.4) while males had an age of 21.5 (95%CI 19.2 to 23.9). Females found to be sterilized at first visit had a mean age of 87.5 at last visit (95%CI 79.3 to 95.7) while males had a mean age of 78.6 (95%CI 70.8 to 86.3) at last visit.

### Population Estimate Compared to Census

**Figure 14:**
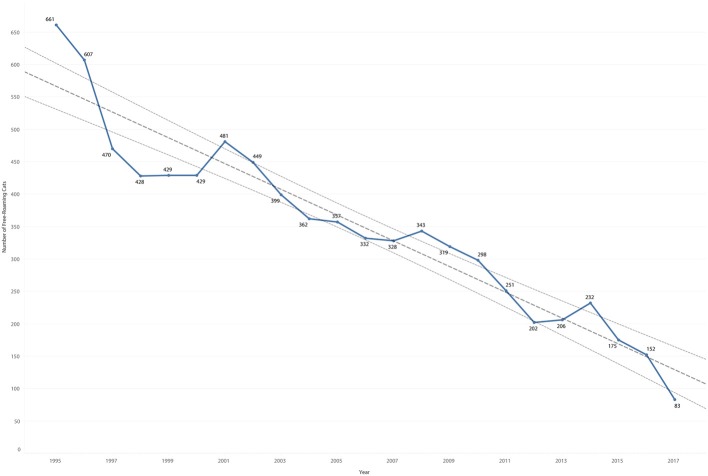
Estimated count of cat population by year based on individual records overlaid by trend line and 95% confidence intervals.

The model of the estimated cat population based on individual records was found to decrease significantly over time (*P* < 0.0001). The decrease was similar to the census values, with comparable slopes (−0.06 for the census, −0.05 for the model). The difference in count per year between the census values and the model for years included in the census ranged from −20 to 30%, with a mean difference of 3.4%. This model estimated the free-roaming population to be 83 in 2017 (Figure 14).

## Discussion

The findings of this study are congruent with prior intensive TNR sites which show a decrease in population over time (2, 16–18). The geographic restriction of this location and duration of the program partially address critiques of previous studies regarding the length of observation and unknown effects of immigration and emigration (12, 19).

Both the observed and modeled population decreased over time, with minor fluctuations observed. The effects of hurricane Irene in October of 1999, Wilma in October of 2005, and Irma in September of 2017, if any, were not discernible with the population data available.^7^ Changes noted in the age structure and modeled population in 2007 and 2013 were due to temporary disruption of the program's trapping efforts. In 2006 the program moved in to the new Grayvik Center, and focus was temporarily diverted from trapping. In 2012 and 2013 there was a temporary change in directorship, which resulted in decreased trapping efforts. The changes observed in the population numbers and age structure subsequent to these two disruptions underscores the importance of continuity in trapping efforts.

Despite the geographically restricted location, there was evidence of a significant amount of introgression (sterilized cats that were not ear tipped), possibly cats brought by seasonal community members or workers that were lost or abandoned or cats from outside geographic areas that were deliberately abandoned. Previously sterilized but not ear-tipped cats most likely represent only 10–20% of lost or abandoned animals, given sterilization rates in at-risk populations (20). The high quality and visibility of the program, which provided food and veterinary care, may have encouraged abandonment of cats if owners believed that the cats would be well taken care of after abandonment. Abandonment may also have occurred if owners believed that cats would be better off under the care of the program rather than surrendered to a shelter where they would face the risk of euthanasia. Interestingly, nine cats sterilized and with ear-tips were noted in the record to not have been sterilized or ear tipped through ORCAT, which suggests deliberate abandonment or, less likely, cats taken to alternative clinic for TNR surgery by an individual. Introgression, particularly of intact cats, has been noted to be a barrier to decreasing cat populations over time through TNR efforts (13, 21, 22). It is unclear whether the introgression observed here was higher or lower than other geographic areas. Access to this location is limited and controlled through a 24-h manned gate, decreasing the likelihood of casual abandonment of cats. It is also geographically isolated, decreasing the chance of cats migrating from adjacent locales. However, human occupation is highly seasonal, which may increase the chance of loss or abandonment by part-time residents and staff. Given the strict control and geographic isolation, required microchipping, sterilization, and licensure of cats might decrease introgression of intact cats.

Retroviral prevalence decreased over time as expected given the elimination of significant risk factors (fighting, mating, vertical transmission) for infection via sterilization, removal of positive cats, and vaccination against FeLV. The point-of-care test that was employed to test for FIV and FeLV is reported to have the best performance for detecting FeLV, with a calculated positive predictive value of 100% for FeLV and between 50 and 84% for FIV depending on prevalence (23). The FeLV vaccine was an adjuvanted killed vaccine that required 2 doses 3–4 weeks apart for efficacy. Because of the inability to safely and humanely house unsocial cats for the duration necessary to booster the vaccine, many cats received only 1 dose. In addition, many cats did not receive recommended re-vaccinations. It is unknown what level of protection may have been afforded from a single FeLV vaccination, and it should be noted that not even fully vaccinated cats are completely protected from infection. For naturally exposed cats, infection with FeLV is approximately 3 times more likely in those unvaccinated as opposed to fully vaccinated (24).

### Limitations

The data are limited as they were collected for programmatic record-keeping rather than epidemiologic analysis. The censuses were not collected at regular intervals, and the years of collection were not regularly spaced. The month of collection was not standard. Cat populations tend to be seasonal, with peak populations observed in the summer and the lowest populations observed in the winter and spring (25). However, neither month nor season were significant in this limited analysis. This may have been due to the preferential removal of juveniles, which make up the vast majority of seasonal variation, or simply a lack of sufficient data points. Classic markers of animal welfare (such as growth, reproduction, body damage, disease, immunosuppression, adrenal activity, behavior anomalies, and self-narcotization) (26) were either not systematically captured or were not captured in a way that could be compared to animals not enrolled in the TNR program and were limited to the measures of life expectancy and a single class of disease prevalence. These measures of cat welfare do not account for concerns regarding return rather than routine euthanasia of trapped cats that include the potential for increased animal suffering due to non-retroviral disease or trauma (in other words, that free-roaming cats would be better off dead).

Another limitation is that all population estimates were counts by a single caretaker. Multiple population census methods would have been ideal, as caretakers may underestimate the number of cats (1). However, this caretaker was highly knowledgeable of the entire population, which she interacted with on a daily basis, which may minimize concerns regarding accuracy of the count. Twenty cats were added to census estimates by the caretaker to account for potential undercounting. The small size of each colony, particularly in later years, should also have made count estimates more accurate.

Nearly all ages were estimates, which makes analysis of age-related data more challenging. The estimated average age of the free-roaming cat population may be biased toward an older age as cats with undocumented removals may have continued to contribute to the average age of the population. This bias was minimized by intensive efforts on the part of ORCAT to document outcomes such as MIA and requests to the community to bring cats that were found dead to the clinic to be outcomed as DOA. Estimated date of death for cats with an outcome of released was based on the average age of death for DOA and euthanized cats, with cats older than that average age at time of release being estimated to live for only an additional 12 months.

In conclusion, a TNR program operating for over two decades achieved a decrease in population and an increase in population welfare as measured by increased average age of population and decreased prevalence of retroviruses.

## Author Contributions

RK collected the data, created the database, entered data, analyzed the data, and was the main author of the manuscript. HC entered data, drafted the introduction of the manuscript and edited the entire manuscript. JL contributed to the study design and data analysis, funded data collection, and edited the manuscript.

### Conflict of Interest Statement

The authors declare that the research was conducted in the absence of any commercial or financial relationships that could be construed as a potential conflict of interest.

## Footnotes

^1^http://www.oceanreef.com

^2^Idexx. SNAP FIV/FeLV Combo Test. Westbrook, Maine, USA.

^3^Esri. 2018. ArcGIS. Redlands, CA, USA.

^4^Filemaker Inc. 2015. Filemaker Pro 14 Advanced. Santa Clara, CA, USA.

^5^Tableau. 2018. Tableau Desktop 2018.1. Seattle, WA, USA.

^6^StataCorp. 2017. Stata Statistical Software: Release 15. College Station, TX, USA.

^7^http://www.hurricanecity.com/city/keylargo.htm
