# Corrigendum: Decrease in Population and Increase in Welfare of Community Cats in a Twenty-Three Year Trap-Neuter-Return Program in Key Largo, FL: The ORCAT Program

**DOI:** 10.3389/fvets.2019.00099

**Published:** 2019-04-02

**Authors:** Rachael E. Kreisler, Heather N. Cornell, Julie K. Levy

**Affiliations:** ^1^Pathology and Population Medicine, Midwestern University College of Veterinary Medicine, Glendale, AZ, United States; ^2^Maddie's Shelter Medicine Program, University of Florida, Gainesville, FL, United States

**Keywords:** trap-neuter-return, TNR, free-roaming cats, feral cats, stray cats, community cats, animal welfare, retrovirus

In the original article, there was an error. The decrease in the free-roaming population was incorrectly stated as “45%” and should be “55%” in both the **Abstract** and **Results**.

A correction has been made to the ***Abstract***:

“The objective of this study was to evaluate the effect of a long-term (23-year) trap-neuter-return program on the population size of community cats in the Ocean Reef Community and to describe the demographic composition and outcome of enrolled cats. A retrospective study was performed using both cat census data collected between 1999 and 2013 as well as individual medical records for cats whose first visit occurred between 3/31/1995 and 12/31/2017. Medical record entries were reviewed to determine program inputs, cat outcomes, retroviral disease prevalence, and average age of first visit, sterilization, and death through 6/11/2018. Change over time was analyzed via linear regression. The free-roaming population decreased from 455 cats recorded in 1999 to 206 recorded in 2013 (55% decrease, *P* < 0.0001). There were 3,487 visits recorded for 2,529 community cats, with 869 ovariohysterectomies and 822 orchiectomies performed. At last recorded visit, there were 1,111 cats returned back to their original location, and 1,419 cats removed via adoption (510), transfer to the adoption center (201), euthanasia of unhealthy or retrovirus positive cats (441), died in care (58), or outcome of dead on arrival (209). The number of first visits per year decreased 80% from 348 in 1995 to 68 in 2017. The estimated average age of the active cat population increased by 0.003 months each year (*P* = 0.031) from 16.6 months in 1995 to 43.8 months in 2017. The mean age of cats at removal increased 1.9 months per year over time (*P* < 0.0001) from 6.4 months in 1995 to 77.3 months in 2017. The mean age of cats at return to the original location was 20.8 months, which did not change over time. The overall retrovirus prevalence over the entire duration was 6.5%, with FIV identified in 3.3% of cats and FeLV identified in 3.6%. Retrovirus prevalence decreased by 0.32% per year (*P* = 0.001), with FIV decreasing by 0.16% per year (*P* = 0.013) and FeLV decreasing 0.18% per year (*P* = 0.033). In conclusion, a trap-neuter-return program operating for over two decades achieved a decrease in population and an increase in population welfare as measured by increased average age of population and decreased retrovirus prevalence.”

As well as the ***Results***, subsection ***Population of Cats***, paragraph one:

“Surveys of the cat population occurred in June 1999, January 2001, March 2003, November 2003, June 2004, June 2006, July 2007, January 2008, July 2009, and February 2013. Per the census records, the free-roaming cat population decreased over time from 455 cats recorded in 1999 to 206 recorded in 2013 (55% decrease). The decrease was linear and significant, with a slope of −0.06, *P* < 0.0001 (Figure 1). Neither month of the year nor a binary seasonal variable of fall/winter as compared to spring/summer were significant.”

Additionally, there was an error in the **Discussion**. An extraneous word (“would”) was erroneously inserted.

A correction has been made to the ***Discussion***, subsection ***Limitations***, paragraph three:

“Nearly all ages were estimates, which makes analysis of age-related data more challenging. The estimated average age of the free-roaming cat population may be biased toward an older age as cats with undocumented removals may have continued to contribute to the average age of the population. This bias was minimized by intensive efforts on the part of ORCAT to document outcomes such as MIA and requests to the community to bring cats that were found dead to the clinic to be outcomed as DOA. Estimated date of death for cats with an outcome of released was based on the average age of death for DOA and euthanized cats, with cats older than that average age at time of release being estimated to live for only an additional 12 months.”

The authors apologize for these errors and state that they do not change the scientific conclusions of the article in any way. The original article has been updated.

